# Applying the eHealth Literacy lens to evaluate a virtual assistant to support family carers of people with dementia

**DOI:** 10.1002/alz.094207

**Published:** 2025-01-09

**Authors:** Tuan Anh Nguyen

**Affiliations:** ^1^ National Ageing Research Institute, Melbourne, VIC Australia

## Abstract

**Background:**

We have co‐produced with carers of people with dementia (hereafter carers) a culturally tailored iSupport Virtual Assistant (VA), namely e‐DiVA, to support English‐, Bahasa‐ and Vietnamese‐speaking carers in Australia, Indonesia, New Zealand and Vietnam. The presented research reports qualitative findings from the e‐DiVA user‐testing study.

**Method:**

Family carers and healthcare professionals working in the field of dementia care were given the e‐DiVA to use on their smartphone or handheld device for 1‐2 weeks. They tested all functions and content of the VA and provided feedback in a ‘Think‐aloud' session, either in person or online. Data was analysed thematically, guided by the eHealth Literacy Framework (eHLF).

**Result:**

Eighteen family carers and nine staff of aged care providers were recruited. Participants discussed all seven domains of eHLF, which were evidenced in the e‐DiVA. They include: 1) ability to process information, i.e. the VA contains information in a format that can easily be understood, e.g. animations; 2) engagement in own health, i.e. providing tailored lessons through need assessments and ‘chatbot'; 3) ability to engage actively with digital services, i.e. providing user manuals; 4) feeling safe and in control; i.e. secure login; 5) motivation to engage with digital services, i.e. providing quick responses/solutions to carers' queries; 6) having access to systems that work, i.e. ability to access to the VA anytime anywhere; and 7) digital services that suit individual needs, i.e. available in users' preferred language. Participants highly ranked domains 1, 2 and 5. They suggested further improvements including making content into audiobooks and messages (domain 1), as well as pointed out some potential barriers to use, especially on domains that require interactions between individuals and the digital system (domains 3, 5 and 7).

**Conclusion:**

Findings show the high acceptance of the VA by both the carers and aged care providers, as well as a need for enhancement to improve accessibility.

